# Pivotal Role of the α_2A_-Adrenoceptor in Producing Inflammation and Organ Injury in a Rat Model of Sepsis

**DOI:** 10.1371/journal.pone.0005504

**Published:** 2009-05-11

**Authors:** Michael Miksa, Padmalaya Das, Mian Zhou, Rongqian Wu, Weifeng Dong, Youxin Ji, Sanna M. Goyert, Thanjavur S. Ravikumar, Ping Wang

**Affiliations:** Center for Immunology and Inflammation, The Feinstein Institute for Medical Research, Department of Surgery, North Shore University Hospital and Long Island Jewish Medical Center, Manhasset, New York, United States of America; University of Giessen Lung Center, Germany

## Abstract

**Background:**

Norepinephrine (NE) modulates the responsiveness of macrophages to proinflammatory stimuli through the activation of adrenergic receptors (ARs). Being part of the stress response, early increases of NE in sepsis sustain adverse systemic inflammatory responses. The intestine is an important source of NE release in the early stage of cecal ligation and puncture (CLP)-induced sepsis in rats, which then stimulates TNF-α production in Kupffer cells (KCs) through the activation of the α_2_-AR. It is important to know which of the three α_2_-AR subtypes (i.e., α_2A_, α_2B_ or α_2C_) is responsible for the upregulation of TNF-α production. The aim of this study was to determine the contribution of α_2A_-AR in this process.

**Methodology/Principal Findings:**

Adult male rats underwent CLP and KCs were isolated 2 h later. Gene expression of α_2A_-AR was determined. In additional experiments, cultured KCs were incubated with NE with or without BRL-44408 maleate, a specific α_2A_-AR antagonist, and intraportal infusion of NE for 2 h with or without BRL-44408 maleate was carried out in normal animals. Finally, the impact of α_2A_-AR activation by NE was investigated under inflammatory conditions (i.e., endotoxemia and CLP). Gene expression of the α_2A_-AR subtype was significantly upregulated after CLP. NE increased the release of TNF-α in cultured KCs, which was specifically inhibited by the α_2A_-AR antagonist BRL-44408. Equally, intraportal NE infusion increased TNF-α gene expression in KCs and plasma TNF-α which was also abrogated by co-administration of BRL-44408. NE also potentiated LPS-induced TNF-α release via the α_2A_-AR in vitro and in vivo. This potentiation of TNF-α release by NE was mediated through the α_2A_-AR coupled Gαi protein and the activation of the p38 MAP kinase. Treatment of septic animals with BRL-44408 suppressed TNF-α, prevented multiple organ injury and significantly improved survival from 45% to 75%.

**Conclusions/Significance:**

Our novel finding is that hyperresponsiveness to α_2_-AR stimulation observed in sepsis is primarily due to an increase in α_2A_-AR expression in KCs. This appears to be in part responsible for the increased proinflammatory response and ensuing organ injury in sepsis. These findings provide important feasibility information for further developing the α_2A_-AR antagonist as a new therapy for sepsis.

## Introduction

Sepsis and septic shock are complications and considered to be major causes of morbidity and mortality in patients with severe trauma, burns, or blood loss [1]. Tissue-fixed macrophages such as the hepatic Kupffer cells (KCs) are involved in inflammatory and metabolic responses to sepsis [2], [3]. The impairment of hepatocellular function observed in early sepsis appears to be due to upregulation of proinflammatory cytokines such as TNF-α [4], [5]. We [6] and Kovarik *et al.* [7] reported that systemic levels of norepinephrine (NE) increased significantly after the onset of sepsis, induced by cecal ligation and puncture (CLP). Enterectomy prior to the onset of sepsis markedly reduced circulating levels of NE, showing that the gut is a major source of NE in sepsis [8]. The catecholamines NE and epinephrine mediate their physiological responses through a group of adrenergic receptor (AR) subtypes [9]. Studies have suggested that NE at concentrations similar to that found in septic animals (∼20 nM) mainly stimulate α_2_-adrenergic receptors (ARs). In our previous studies, we reported that gut-derived NE upregulates TNF-α production in KCs through the α_2_-adrenergic pathway [9]. This is a novel finding, since the immunological effect of the sympathetic nerve activity and the adrenal epinephrine was previously considered to be anti-inflammatory through the activation of β-ARs on leukocytes [10]. α_2_-ARs are G-protein coupled receptors that mediate the central and peripheral actions of the primary sympathetic neurotransmitter, NE and the adrenal hormone epinephrine through the intracellular suppression of cAMP [11]. However, it remains unknown which of the three α_2_-AR subtypes (i.e., α_2A_, α_2B_ or α_2C_) is responsible for the upregulation of TNF-α production. The aim of this study was therefore to determine the contribution of α_2A_-AR in NE-mediated proinflammatory effects in sepsis.

## Materials and Methods

### Experimental model of sepsis

Polymicrobial sepsis was induced in adult male rats by cecal ligation and puncture (CLP). Briefly, rats were fasted overnight prior to the induction of sepsis, but allowed water *ad libitum.* The animals were anesthetized with isoflurane inhalation and a 2-cm ventral midline abdominal incision was made. The cecum was then exposed, ligated just distal to the ileocecal valve to avoid intestinal obstruction, punctured twice with an 18-gauge needle, and returned to the abdominal cavity. The incision was closed in layers and the animals were resuscitated with 3 ml/100 g BW normal saline subcutaneously immediately after CLP [12]. This model of sepsis is associated with an early, hyperdynamic phase (i.e., 2–10 h after CLP; characterized by an increased cardiac output and tissue perfusion, decreased vascular resistance, and hyperglycemia), which is followed by a late, hypodynamic phase (16 h after CLP and later; characterized by reduced cardiac output and tissue perfusion, increased vascular resistance, and hypoglycemia) [5], [13], [14]. Sham-operated animals underwent the same surgical procedure except that the cecum was neither ligated nor punctured. Studies were then conducted at 2 h (early sepsis) and 20 h (late sepsis) after the induction of sepsis. This project was approved by the Animal Care and Use Committee of the Feinstein Institute for Medical Research and following national guidelines for the use of animals in research.

### Isolation of Kupffer cells

Kupffer cells were isolated from normal and septic rats as previously described elsewhere with some modifications [12]. Briefly, under isoflurane anesthesia, following a midline incision the inferior vena cava was cannulated and the portal vein was severed. The liver was immediately perfused in situ with ∼60 ml of Hanks balanced salt solution without Ca^2+^ and Mg^2+^ (Cellgro, VA) at 37°C at a rate of 15 ml/min. This was followed by perfusion with 120 ml of HBSS containing 0.02% collagenase (Worthington, Lakewood, NJ; Type IV, 180 U/mg) and 100 mM CaCl_2_ solution at the same perfusion rate. The liver was then removed en bloc, rinsed with ∼25 ml of HBSS, minced in a petridish containing HBSS with collagenase, and incubated for 20 min at 37°C to further dissociate the cells. The cell suspension was then passed through a 150-mesh, stainless steel screen into cold Dulbecco modified Eagel medium (DMEM; GIBCO Life Technologies, Carlsbad, CA), containing 10% heat-inactivated fetal bovine serum and centrifuged (50 g for 2 min at 4°C) to sediment hepatocytes. The remaining cells in the supernatant were collected by centrifugation (450 g for 10 min at 4°C). The cell pellets resuspended in DMEM. After washing twice, cells were centrifuged on a density cushion of Percoll at 1,000 g for 15 min at 4°C. The buffy coat containing the KCs fraction was collected. The cells were further washed twice. Cell viability as determined by trypan blue exclusion was more than 95%. The yield was at 8–12×10^6^ KCs/liver with a purity of at least 90%. The isolated KCs were then cultured in DMEM, containing 10% heat-inactivated fetal bovine serum, 10 mM HEPES, 100 U/ml penicillin and 100 g/ml streptomycin at the concentration of 10^6^ cells/ml overnight with 5% CO_2_ at 37°C. KCs were allowed to adhere to the bottom of a 24-well plate (Costar) overnight and unattached cells were removed by gentle washing.

### Assessment of α_2A_-AR mRNA

Gene expression of α_2A_-AR was assessed by real-time quantitative PCR (Q-PCR). Total RNA was extracted from KCs of CLP and Sham-operated animals as well as from cultured KCs using Tri-reagent (Molecular Research Center, Cincinnati, OH). Q-PCR was carried out on cDNA samples reverse transcribed from 2 µg RNA using murine leukemia virus reverse transcriptase (Applied Biosystems). Using the QuantiTect SYBR Green PCR kit (Qiagen, Valencia, CA), reactions were carried out in 24 µl final volume containing 2 pmol of forward and reverse primers, 12 µl QuantiTect Master Mix, and 1 µl cDNA. Amplification was performed according to Qiagen's recommendations with an Applied Biosystems 7300 real-time PCR. Expression amount of rat GAPDH mRNA was used for normalization of each sample, and analysis of each specific mRNA was conducted in duplicate. Relative expression of mRNA was calculated by the ΔΔ*Ct*-method, and results expressed as fold change with respect to the corresponding experimental control. The following rat primers were used: GAPDH (AF 106860): 5′-ATG ACT CTA CCC ACG GCA AG-3′ (forward), 5′-CTG GAA GAT GGT GAT GGG TT-3′ (reverse); rat α_2A_-AR (NM_012739), 5′-CGT GTT CGT GGT GTG TTG GT-3′ (forward), 5′-GCA GCC GAC CGC TAT GAG-3′ (reverse).

### Binding capacity and affinity of KC α_2_-adrenoceptors

Freshly isolated KCs (10^6^) from sham and septic animals at 2 h after CLP were incubated with [^3^H]-yohimbine (a radioactively labeled α_2_-AR antagonist; specific activity 79.2 Ci/mmol; Dupont/NEN; final concentration, 2 to 64 nM in a volume of 200 µl) with or without 10 µM of unlabeled yohimbine for 30 min at 37°C in an assay buffer (40 mM Tris-HCl, 10 mM MgCl_2_, pH 7.5) [15]. The value of B_max_ and K_d_ were determined by Scatchard analysis after logarithmic transformation.

### Stimulation of isolated Kupffer cells with the α_2A_-AR subtype inhibitor BRL-44408

KCs isolated from normal animals were cultured overnight in DMEM medium with 10% heat inactive fetal calf serum, 100 U/ml penicillin/streptomycin, 100 mM HEPES and 100 U/ml L-glutamine at the concentration of 10^6^ cells/ml. KCs were then stimulated with NE (20 nM) with or without α_2A_-AR specific antagonist BRL-44408 maleate (1 µM, Tocris, UK) for 4 h. The supernatant was then collected and TNF-α levels were measured by enzyme-linked immunosorbent assay (ELISA) kit specific for rat TNF-α (Pharmingen, San Diego, CA). The assay was carried out according to the instructions provided by the manufacturer. For additional p38 MAP kinase pathway studies, KCs were cultured in DMEM for an 1 h (p38 phosphorylation) or 24 h (TNF-α release) with the following treatments: NE (20 nM), LPS (100 ng/ml, *E. coli* 055:B5; Sigma, St. Louis, MO), and the inhibitors BRL-44408 (1 µM), pertussis toxin (PTX, 100 ng/ml), or SB203580 (10 µM).

### Intraportal administration of NE

Following anesthesia with isoflurane, a 3-cm midline incision was performed. The small intestine was exposed and a branch of the superior mesenteric vein was cannulated with a PE-10 catheter. It should be noted that this procedure did not cause any apparent gut ischemia. NE (20 µM in normal saline containing 0.1% ascorbic acid to prevent NE oxidation) or vehicle was infused into the portal vein at a rate of 13 µl/min for 2 h using a Harvard pump. Since portal blood flow is ∼13 ml/min/liver [16], the above rate of NE infusion would be expected to increase the portal NE level to 20 nM, which is similar to that observed during sepsis[6]. A third group also received BRL-44408 maleate (1 mM solution at 13 µl/min), which was first infused into the portal vein for 15 min and then followed by infusion of 20 µM NE in combination with 1 mM BRL-44408 for 2 h at an infusion rate of 13 µl/min. After 2 h blood samples were collected by cardiac puncture and KCs were isolated as described above. In additional groups of NE, NE plus BRL-44408, or vehicle-infused animals, LPS (7.5 mg/kg) was administered through intra-peritoneal injection at 30 min after the onset of 2-h infusion. At the end of the infusion (i.e., 1.5 h after LPS challenge), blood samples were collected for plasma TNF-α measurement.

### Determination of TNF-α production

Plasma, supernatant from KC culture, and cellular (5×10^6^ KCs) TNF-α levels were determined using an ELISA assay kit specific for rat TNF-α (Pharmingen, San Diego, CA). Isolated KCs were also used to determine the TNF-α gene expression by RT-PCR as described previously [17].

### Determination of p38 MAP kinase phosphorylation

KCs were lysed (10 mM Tris saline, pH 7.5 with 1% Triton-100×, 1 mM EDTA, 1 mM EGTA, 2 mM Na-orthovanadate, 0.2 mM PMSF, 2 µg/ml leupeptin, 2 µg/ml aprotinin), centrifuged at 16,000 g for 10 min, the supernatant was collected, and the protein concentration determined. A total of 10 µg of protein was loaded on a 4–12% Bis-Tris gel (Invitrogen, Carlsbad, CA) and electrophoretically fractionated in a MES SDS running buffer (Invitrogen). The protein was then transferred to a 0.45-µm nitrocellulose membrane, and blocked with 5% bovine serum albumin in 10 mM Tris saline with 0.1% Tween 20, pH 7.6 (TBST). The membrane was incubated with rabbit anti-phosphorylated p38 MAP kinases polyclonal antibodies (1∶1000) overnight at 4°C, followed by incubation in 1∶20,000 HRP-linked anti-rabbit IgG for 1 h at room temperature. To reveal the reaction bands, the membrane was reacted with ECL Western blot detection system and exposed on X-ray film. The same membranes were stripped and re-blotted with rabbit anti-total-p38 MAP kinases (1∶500; please note that stripping and re-blotting may have reduced the signals) to determine the ratio of phospho-p38 and total p38. A digital image system was used to determine the density of the bands (Bio-Rad, Hercules, CA).

### Treatment of septic rats with BRL-44408

Rats underwent CLP as described before, the femoral vein was canulated and connected to a Harvard pump. BRL-44408 (2.5 mg/kg BW) or vehicle was infused for 30 minutes using a Harvard pump. The catheter was tunneled out, and the femoral vein was ligated to stop the bleeding. The animals were allowed to wake up and returned to their cages. 20 h later animals sacrificed and blood collected for the measurement of liver transaminases, creatinine, lactate and TNF-α using commercial assay kits (Pointe Scientific). KC TNF-α mRNA expression was also assessed using the methods described above. The liver enzymes AST and ALT as well as creatinine and lactate levels were measured in blood serum by using commercial assay kits (Pointe Scientific, Canton, MI). In addition we conducted a 10-day survival study was conducted. 20 animals in each group underwent CLP and received either BRL-44408 (2.5 mg/kg BW bolus iv) or the same volume normal saline (Vehicle). 20 h after CLP, the cecum was removed, which based on our experience normally yields a 10-day survival rate of approximately 50%. In additional groups of normal rats, both the left femoral vein and artery were cannulated with a PE-50 tubing. The femoral vein catheter was connected to a Harvard pump for BRL-44408 (2.5 mg/kg BW) or vehicle infusion as described above. The femoral artery catheter was connected to a blood pressure analyzer (BPA; Digi-Med, Louisville, KY) and mean arterial pressure (MAP) and heart rate were recorded continuously.

### Statistical analysis

All data that passed the normality test are expressed as mean±SE and compared by Student's t-test or analysis of variance (ANOVA) using Student-Newman-Keul's post-hoc analysis. Data that are of percentile based nature or failed the normality test are expressed as mean±95% confidence, and compared with ANOVA on Ranks. The binding capacity and affinity was estimated using the Scatchard analysis and curves compared by two-way ANOVA. The survival study was analyzed using the Kaplan-Meyer log-rank test. Differences in values were considered significant if P<0.05. All statistical analysis was performed using either the SigmaStat® or PRISM® software.

## Results

### Upregulation of KC α_2A_-AR expression in CLP-induced sepsis

**Figure 1:**
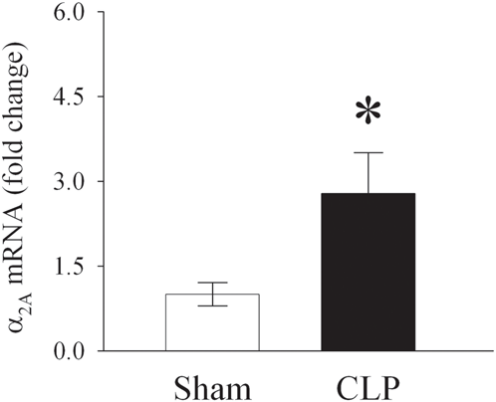
Upregulation of KC α_2A_-AR expression in CLP-induced sepsis. Gene expression of α_2A_-adrenergic receptor (AR) in Kupffer cells isolated from animals at 2 h after cecal ligation and puncture (CLP) or sham-operation. Relative expression of mRNA was calculated by the ΔΔ*Ct*-method, and results expressed as fold change with respect to the housekeeping gene GAPDH. Values (n = 6/group) are presented as mean±SE and compared by Student's *t*-test. *P<0.05 vs. Sham.

Rats were subjected to sepsis by CLP and KCs were isolated 2 h thereafter. As shown in Figure 1, the gene expression of α_2A_-AR was significantly upregulated by 179% at 2 h post-CLP compared to respective sham-operated animals. In contrast, α_2B_ and α_2C_-AR expression did not show any changes after CLP (Data not shown).

### Increased KC α_2_-AR binding capacity and affinity in sepsis

**Figure 2:**
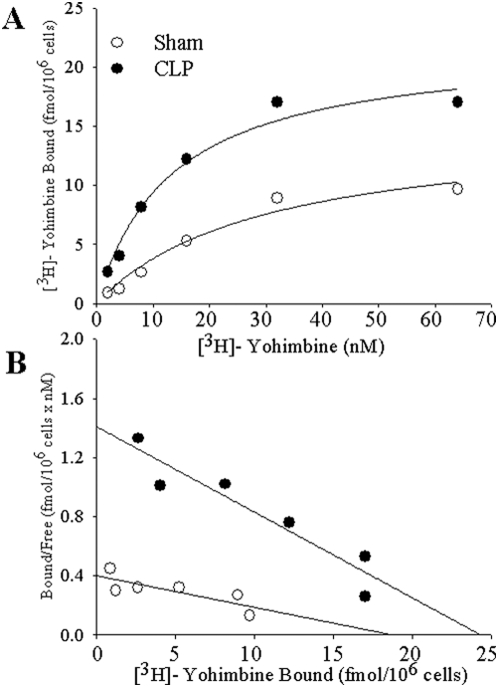
Increased KC α_2_-AR binding capacity and affinity in sepsis. Changes in α_2_-receptor binding of the radiolabeled specific ligand [^3^H]-yohimbine in rat Kupffer cells obtained from sham-operated and cecal ligation and puncture (CLP) animals (A). B_max_ and K_d_ were estimated using the the Scatchard analysis (B). The data points represent the average of three different experiments. Best fit analysis and two-way ANOVA showed that curves are different for Sham and CLP (P = 0.0036).

To investigate whether increased α_2_-AR expression in sepsis leads to enhanced receptor binding, we incubated KCs isolated from sham-operated or CLP animals with [^3^H]-yohimbine, a radio-labeled α_2_-specific AR antagonist. As shown in Figure 2A, the binding of KC α_2_-AR was saturated at approximately 30 nM [^3^H] yohimbine in both sham-operated and CLP animals. However, maximal binding of the α_2_-specific ligand was much higher in KCs from CLP animals (**Fig. 2A**). Data transformation to a Scatchard plot yielded linear regression lines consistent with a single class of antagonist binding capacity (**Fig. 2B**). Scatchard analysis revealed a 28% increase in maximal binding capacity with an average 24.3 fmol/10^6^ cells 2 h after CLP compared to a B_max_ of 19.0 fmol/10^6^ cells in sham animals (**Fig. 2B**). Similarly, the average K_d_ decreased by 64% from 47.6 nM in sham animals to 17.2 nM 2 h after CLP (**Fig. 2B**), indicating increased affinity in septic animals. In addition, we discovered a 50% reduction in cAMP levels in KCs that decreased from 7.31±0.14 pmol/5×10^6^ cells in sham-operated animals to 3.82±1.70 pmol/5×10^6^ cells at 2 h after CLP (decreased by 48%, n = 4–5; P<0.05).

### Stimulation with NE increases TNF-α release from KCs via α_2A_-ARs

**Figure 3:**
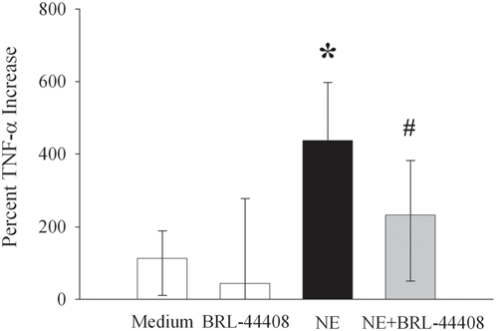
Stimulation with NE increases TNF-α release from KCs via α_2A_-ARs. Alterations of TNF-α after stimulation of isolated KCs with NE (20 nM) with or without BRL-44408 maleate (1 µM), a specific α_2A_-AR antagonist, for 4 h. Percentage values (n = 6/group) are presented as mean±95% confidence and compared by ANOVA on Ranks and Student-Newman-Keul's method. *P<0.05 vs. Medium; #P<0.05 vs. NE.

Isolated KCs were stimulated with NE (20 nM) with or without BRL-44408 maleate (1 µM) for 4 h. While TNF-α release increased by 440% after NE-stimulation, its increase was completely suppressed after α_2A_-AR blockade by BRL-44408 maleate (**Fig. 3**). BRL-44408 alone did not have any measurable effects on TNF-α release in the absence of NE-stimulation (**Fig. 3**).

### Portal infusion of NE induces α_2A_-AR-dependent TNF-α production in KCs

**Figure 4:**
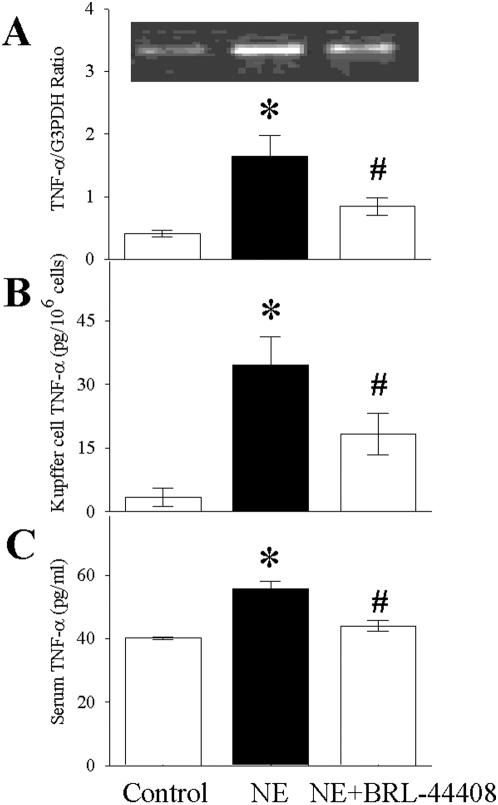
Portal infusion of NE induces α_2A_-AR-dependent TNF-α production in KCs. *In vivo* Alterations in Kupffer cell gene expression (A) and protein levels of TNF-α (B) as well as serum TNF-α levels (C) after administration of NE, NE combined with BRL-44408 maleate for 2 h through the portal vein. A representative gene and ratio of TNF-α and housekeeping gene G3PDH are presented in panel (A). Values (n = 5/group) are presented as mean±SE and compared by one-way ANOVA and Student-Newman-Keul's method. *P<0.05 vs. Control; #P<0.05 vs. NE.

To elucidate whether the α_2A_-AR is also responsible for TNF-α upregulation in vivo, we administered NE through the portal vein in normal animals for 2 h and isolated KCs for analysis. TNF-α gene expression was upregulated by 4-fold in KCs from animals that were subjected to intraportal infusion of NE as compared to vehicle-treated animals (Fig. 4A). BRL-44408 pretreatment prevented the upregulation of TNF-α gene expression. Similarly, TNF-α protein levels increased after intraportal infusion of NE by 10-fold and co-administration of NE with BRL-44408 maleate reduced cellular TNF-α levels by 47% (Fig. 4B). Serum levels of TNF-α also increased after intraportal infusion of NE from 40.2±0.8 pg/ml to 55.7±5.2 pg/ml (Fig. 4C). BRL-44408 significantly suppressed plasma TNF-α levels by 25% to blood TNF-α concentrations found in sham operated animals (Fig 4C). These results underline the crucial role of the α_2A_-AR in the proinflammatory response of Kupffer cells after NE-stimulation under in vivo conditions.

### NE-mediated potentiation of LPS-mediated TNF-α release through the α_2A_-AR

**Figure 5:**
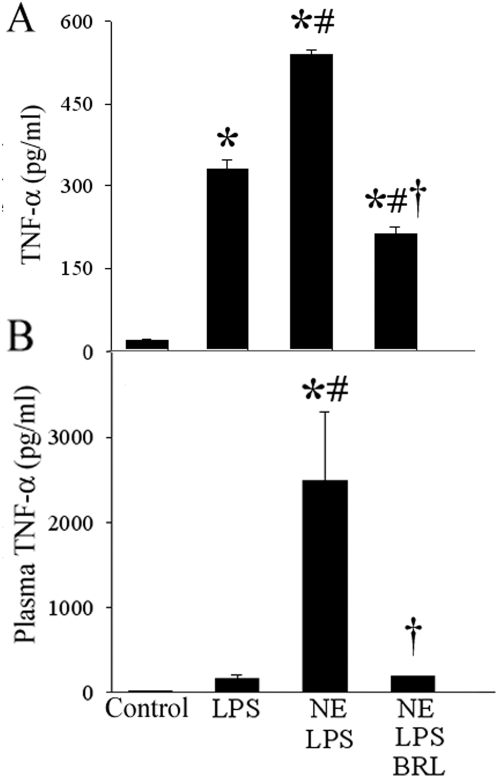
NE-mediated potentiation of LPS-mediated TNF-α release through the α_2A_-AR. BRL-44408 maleate blocks TNF-α production in LPS-stimulated KCs after co-incubation with NE *in vitro* and *in vivo*. (A) Alterations in TNF-α release from cultured KCs 24 h after stimulation with NE (20 nM) and LPS (100 ng/ml), with or without BRL-44408 (BRL, 1 µM). Data are presented as mean±SE (n = 8) and compared by one-way ANOVA and Student-Newman-Keuls test. *P<0.05 vs. Control; #P<0.05 vs. LPS alone; †P<0.05 vs. NE+LPS. (B) Alterations in plasma levels of TNF-α after administration of LPS (7.5 mg/kg, intra-peritoneal) and NE (20 µM NE for 2 h at 13 µl/min), with or without BRL-44408 (2.5 mg/kg BW, intra-portal). Data are presented as mean±SE (n = 4–6) and compared by one-way ANOVA and Student-Newman-Keul's method. *P<0.05 vs. Control; #P<0.05 vs. LPS alone; †P<0.05 vs. NE+LPS.

To investigate the role of the α_2A_-AR in NE-mediated potentiation of a proinflammatory response to endotoxin, we studied the response of KCs to NE and LPS in the presence or absence of the α_2A_-specific inhibitor BRL-44408 in vitro (Fig. 5A) and in vivo (Fig. 5B). Cultured KCs were responsive to endotoxin and TNF-α levels increased by over 16-fold after stimulation with 100 ng/ml LPS (P<0.05, Fig. 5A). The simultaneous treatment with NE+LPS caused TNF-α levels increased by an additional 63% (P<0.05 vs. LPS alone, Fig. 5A), indicating a potentiation of LPS-induced TNF-α release from cultured KC. Concurrent inhibition of the α_2A_-AR using BRL-44408 (1 µM) significantly attenuated TNF-α release by over 60% (Fig. 5A). To verify the crucial role of the α_2A_-AR in the proinflammatory response to NE in endotoxemia, we measured plasma levels of TNF-α after intraperitoneal injection of LPS (7.5 mg/kg) and systemic intravenous administration of NE. LPS alone resulted in a 10-fold increase of TNF-α plasma levels after 4 h (Fig. 5B) and the co-administration of NE resulted a marked enhancement in plasma TNF-α release by 155-fold (P<0.05; Fig. 5B). Administration of the α_2A_-AR antagonist BRL-44408 completely blocked the LPS/NE-induced TNF-α release (Fig. 5B). Thus, NE-mediated potentiation of LPS-induced TNF-α release is α_2A_-AR-dependent even after systemic application of NE.

### α_2A_-AR-dependent activation of p38 MAP kinase

**Figure 6:**
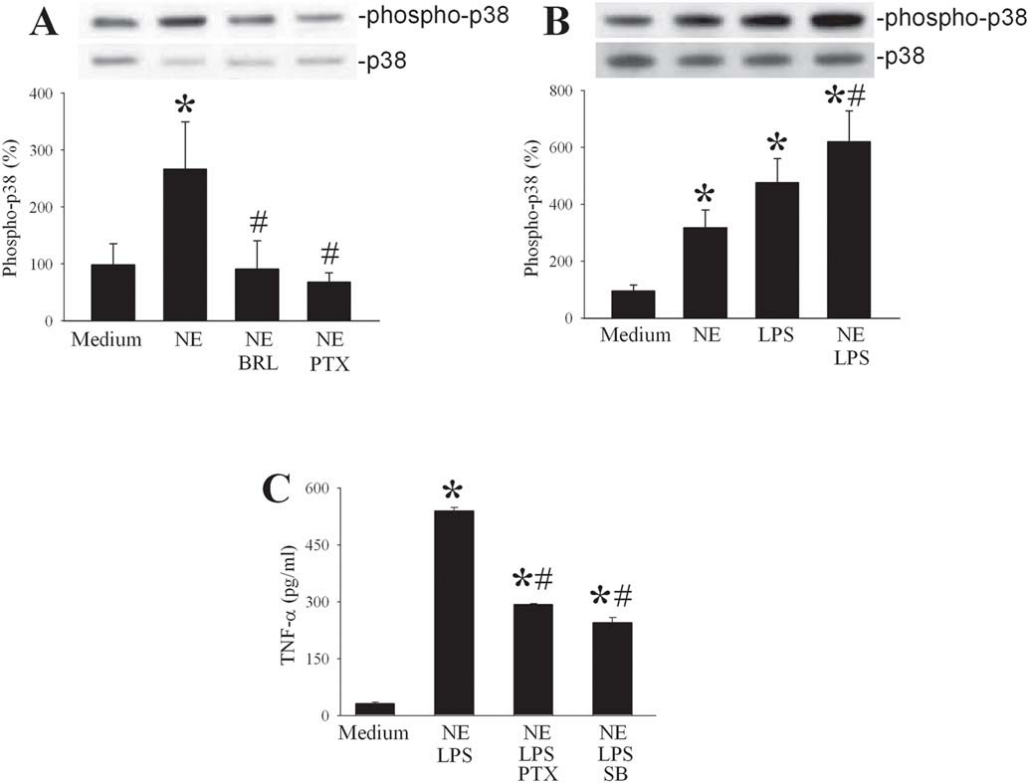
NE-mediated potentiation of p38 MAP kinase phosphorylation via α_2A_-ARs. (A) Alterations in p38 MAP kinase after 1 h culture with NE alone (20 nM), NE+BRL-44408 (BRL, 1 µM), or NE+pertussis toxin (PTX, 100 ng/ml). The relative percentage of phosphorylated p38/total p38 MAP kinase is presented as mean±95% confidence (n = 4–6) and compared by ANOVA on Ranks and Student-Newman-Keul's method. The Medium group is considered as 100%. *P<0.05 vs. Medium; #P<0.05 vs. NE alone. Representative gels are presented. (B) Enhanced activation of p38 MAP kinase after 1 h culture with a combination of NE (20 nM) and LPS (100 ng/ml). The relative percentage of phosphorylated p38/total p38 MAP kinase ratio is presented as mean±95% confidence (n = 4–6) and compared by ANOVA on Ranks and Student-Newman-Keul's method. Medium group is considered as 100%. *P<0.05 vs. Medium; #P<0.05 vs. LPS alone. Representative gels are also presented. (C) Suppression of TNF-α release from cultured KCs after stimulation with NE (20 nM)+LPS (100 ng/ml) with or without pertussistoxin (PTX, 100 ng/ml), a G_αi_-protein inhibitor, or SB203580 (10 µM), a p38 MAP kinase inhibitor. Data are presented as mean±SE (n = 4–6) and compared by one-way ANOVA and Student-Newman-Keul's method. *P<0.05 vs. Medium; #P<0.05 vs. LPS+NE.

LPS induces the activation of intracellular pathways, including the p38 MAP kinase by its phosphoryation, which plays a crucial role in the proinflammatory response of macrophages [18]. To determine, whether α_2A_-AR activation affects the p38 pathway, we stimulated cultured KCs with NE (20 nM) and blocked either the α_2A_-AR with BRL-44408 or its coupled G_αi_-protein with pertussis toxin (PTX). Both BRL-44408 (1 µM) and pertussis toxin (PTX, 100 ng/ml) prevented the NE-induced phosphorylation of the p38 MAP kinase (**Fig. 6A**). This indicates that NE acts through the α_2A_-AR and G_αi_ protein to activate the p38 MAP kinase. To verify this effect under inflammatory conditions, we assessed NE-mediated phosphorylation of the p38 MAP kinase in cultured KC at 1 h after stimulation with LPS (100 ng/ml). Either agent by itself induced the phosphorylation of p38 compared to control, however, the combination of NE and LPS showed the strongest activation (over 7-fold induction) of p38 in cultured KCs (**Fig. 6B**). This indicates that LPS-induced p38-activation can be potentiated by simultaneous NE stimulation. To determine the role of this pathway in the α_2A_-AR-mediated proinflammatory effect of NE, additional experiments were performed to measure TNF-α release after NE+LPS stimulation with or without inhibition of the G_αi_ or the p38 MAP kinase. Both PTX (100 ng/ml) and SB203580 (10 µM; a p38 MAP kinase inhibitor) attenuated the NE-mediated increase in TNF-α release by 46% and 55% respectively (**Fig. 6C**). This study further confirms that NE potentiates LPS-induced TNF-α through the α_2A_-AR coupled G_αi_ protein and the activation of the p38 MAP kinase.

### BRL-44408 is beneficial in experimental sepsis

**Figure 7:**
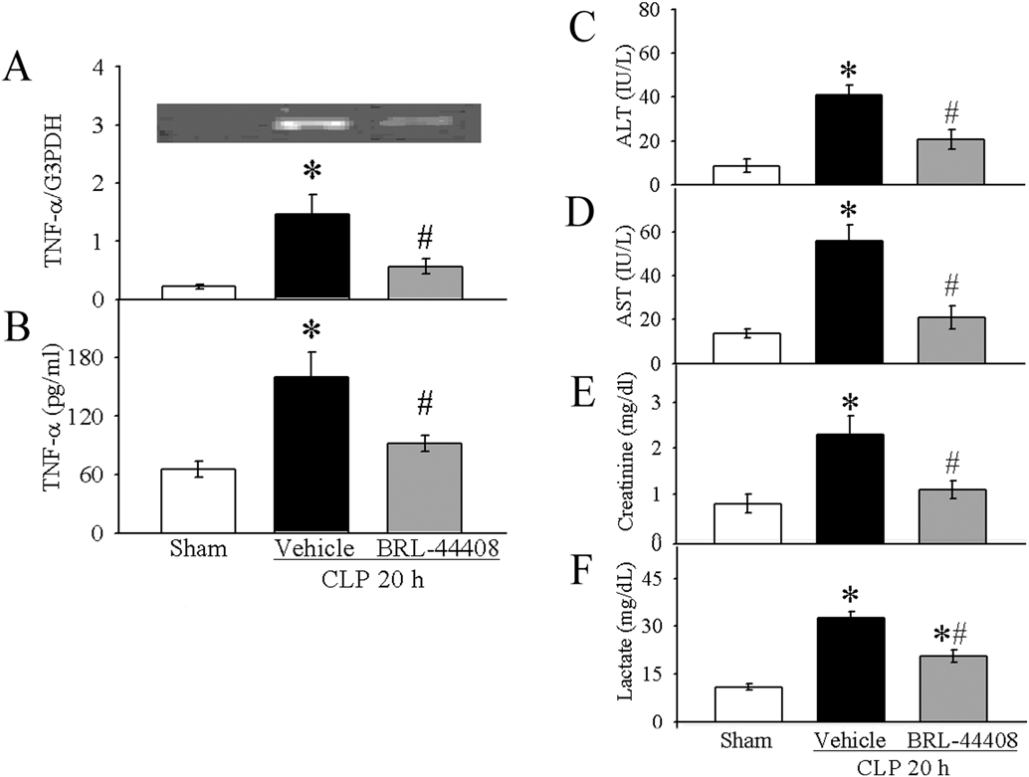
Beneficial effects of α_2A_-AR inhibition in sepsis. (A–B) BRL-44408 mediated suppression of TNF-α gene expression in Kupffer cells (A) and plasma concentrations (B) 20 h after CLP. (C–F) BRL-44408 mediated improvement of organ damage parameters. Rats underwent CLP and 20 h later ALT (C), AST (D), creatinine (E) and lactate (F) levels were measured as described in the methods. Values (n = 5/group) are presented as means±SE and compared by one-way ANOVA and Student-Newman-Keul's method. *P<0.05 vs. Sham; #P<0.05 vs. CLP+Vehicle.

**Figure 8:**
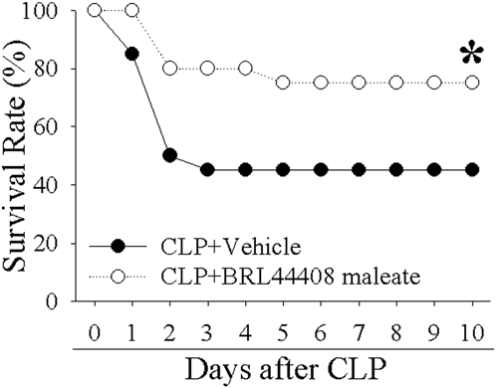
The α_2A_-AR inhibitor BRL-44408 improves survival in septic rats. Rats underwent CLP (n = 20/group) and received either Vehicle treatment or BRL-44408 maleate iv 2.5 mg/kg BW. Cecums were removed 20 h later and animals observed for up to 10 days. *P<0.05 vs. Vehicle, Kaplan-Meyer logrank test.

**Figure 9:**
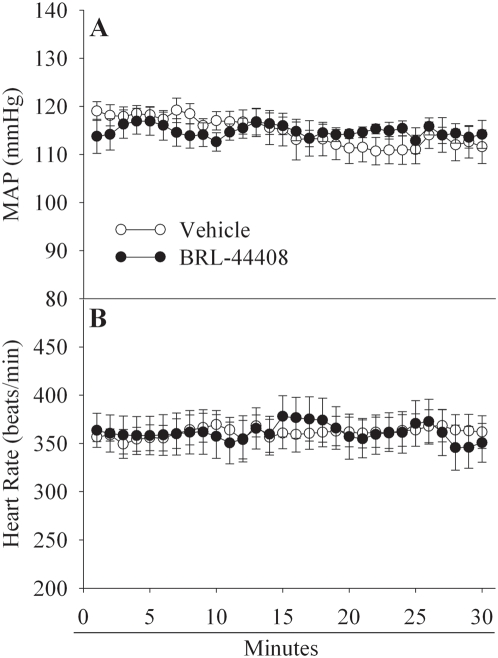
BRL-44408 has no effects on MAP and heart rates in normal rats. Effects of α_2A_-AR inhibitor BRL-44408 on mean arterial pressure (MAP, A) and heart rates (B) in normal rats. Normal rats received either Vehicle treatment or BRL-44408 maleate iv 2.5 mg/kg BW over a period of 30 min. Data are presented as mean±SE (n = 5/group), and compared by Student's *t*-test. No statistical difference was found.

Using the above *in vitro* and *in vivo* systems, we were able to show that BRL-44408 can attenuate the proinflammatory effect of NE either alone or in conjunction with LPS. To investigate the beneficial effect of BRL-44408 in sepsis, we used an experimental sepsis model of CLP in rats receiving BRL-44408. 20 h later we measured cytokine levels in KCs, plasma and injury paramers for liver (AST & ALT), kidney (creatinine) and general oxigenation (lactate). As expected, TNF-α mRNA levels in KCs as well as plasma levels were increased 20 h after CLP by 6.6-fold and 2.4-fold, respectively (**Figs. 7A–B**). BRL-44408 treatment completely inhibited TNF-α production and release (**Figs. 7A–B**). Similarly, CLP-induced increases in surrogate markers for liver and kidney injury (AST, ALT and creatinine by 4.8-, 4.1-, and 2.9-fold, respectively) were completely blocked after treatment with BRL-44408 (**Figs. 7C–E**). Lactate, a marker for tissue perfusion and oxygenation, that was increased by 3-fold after CLP and was significantly suppressed by 37% after BRL-44408 infusion (**Fig. 7F**). These above results indicate that BRL-44408 confers an anti-inflammatory effect and protects from organ injury and tissue malperfusion in CLP-induced sepsis in rats. To show that these beneficial effects translate into an improved outcome, we conducted a survival study. As shown in **Figure 8**, CLP and vehicle treatment resulted in a 55% mortality rate over a 10-day period. Treatment with BRL-44408, however, protected over 55% of animals at risk, resulting in an overall survival rate of 75%. To determine the effect of the α_2A_-AR blockade on other organ system, we monitored MAP and heart rates during BRL-44408 administration in normal rats. As shown in **Figure 9**, intravenously infusion of BRL-44408 at the dose of 2.5 mg/kg BW had no measurable effects on MAP and heart rates.

## Discussion

Gut-derived NE has been shown to play a critical role in inducing hepatocellular dysfunction in early sepsis, exerting its effect through the non-synaptic, high-affinity α_2_-AR [19]. Kotanidou *et al*. have shown that urethane, a general anesthetic with α_2_-AR blocking properties, protects against LPS partly by reducing TNF-α release [20]. We have previously reported that TNF-α secretion from NE stimulated Kupffer cells can be inhibited by the general α_2_-AR inhibitor yohimbine, suggesting that α_2_-ARs on Kupffer cells are particularly responsible for the upregulation of TNF-α release [21]. The presence of α_2_-ARs on macrophages has been previously confirmed by receptor binding assays and *in situ* hybridization [22], [23]. Studies by other groups have shown that TNF-α upregulation can be mediated by the stimulation of α_2_-ARs [11], [24]. Here we show that it is the α_2A_-AR that is upregulated in KCs leading to enhanced receptor binding and proinflammatory cytokine release in sepsis.

We have previously shown that TNF-α is significantly increased by incubation with NE (20 nM) for 4 h [21]. Since KCs are a major source of proinflammatory cytokines [25], intraportal infusion of NE appears to have a direct measurable effect on TNF-α release *in vivo*. Infusion of NE through the femoral vein may reduce active NE levels reaching the liver compared to direct intraportal injection. Hence, one may expect a diminished proinflammatory response of NE after systemic administration. We have shown however, that even after peripheral intravenous administration of NE, the LPS-induced TNF-α increase becomes dramatically potentiated through an α_2A_-AR-dependent pathway, possibly through the involvement of other tissue macrophages.

Adrenergic receptors are subdivided in to three major subtypes α_1_, α_2_, and β, which are then subdivided into α_1A_, α_1B_, and α_1C_, α_2A_, α_2B_, and α_2C_ and β_1_, β_2_, and β_3_. Upon binding of β_2_-ARs for example, epinephrine and high doses of NE through increasing intracellular cAMP levels [26]. α_2_-adrenoceptors are G_i_- and G_0_-protein coupled receptors that decrease intracellular cAMP, open K^+^ channels, and inhibit voltage gated Ca^2+^ channels, all of which lead to hyperpolarization of neurons and activation of immune cells [26]. In the CNS, α_2_-adrenoceptors are predominantly presynaptic. They regulate the release of neurotransmitters through a negative feedback. Functional studies of the genetic receptor subtypes have linked the α_2B_-adrenoceptor to peripheral vasoconstriction and analgesic effects of N_2_O (nitrous oxide) and other anesthetic agents. The α_2A_-AR, either alone or with α_2C_-AR co-activation, is involved in the central inhibition of sympathetic activities, modulation of neurotransmitter release, sedation, and anti-epileptic effects. As we have shown here, the pro-inflammatory action of NE, mediated by the α_2A_-receptor subtype expressed on hepatic macrophages (i.e., KCs) can now be added to this list.

So far, however, we could find only one report regarding the α_2_-AR subtypes responsible for TNF-α upregulation in pulmonary inflammation and none in sepsis itself. In their work by Flierl *et al.*, the authors focused on the phagocyte-derived cathecholamines that boost inflammatory responses via the α_2_-AR [27]. Although this report indicated similar increases in the α_2A_-AR subtype in alveolar macrophages and neutrophils after LPS-stimulation, the role of individual subtypes in the proinflammatory response was not addressed [27]. In our study we have assessed the influence of these subtypes in hepatic macrophages, i.e., KCs. We have shown that the gene expression of α_2A_-AR has significantly increased 2 h after CLP, while no significant changes in α_2B_ and α_2C_-AR could be observed. Kupffer cells stimulated with NE in combination with the α_2A_-AR inhibitor BRL-44408 maleate reduced TNF-α release, while the α_2B_-AR inhibitor imiloxan hydrochloride increased TNF-α levels (data not shown). The difference between those two receptor subtypes may lie in the intracellular signaling pathways. While all α_2_-ARs suppress intracellular cAMP levels through its G_αi_ coupled protein, they also change intracellular calcium and potassium levels to a different degree, which may influence intricate signaling pathways and eventually cellular response. As opposed to *in vivo* findings that showed no differences, our *in vitro* results show that α_2B_-AR inhibition is able to inhibit TNF-α release from cultured KCs, which is further complicating the role of α_2B_-ARs in the inflammatory response. *In vivo*, intraportal administration of NE significantly increased serum and Kupffer cell levels of TNF-α, and only the α_2A_-AR specific antagonist, BRL-44408 could significantly reduce TNF-α plasma levels and Kupffer cell TNF-α release. Recent reports show that not only sympathetic mediators, but also the cholinergic pathways modulate the systemic inflammatory response. Thus TNF-α release can be reduced by increasing efferent vagus nerve activity and acetylcholine release [28]. In this regard, the nicotinic acetylcholine receptor α_7_ subunit appears to be responsible for the inhibition of macrophage TNF-α release by acetylcholine [29]. The role of the α_2A_-AR in the regulation of vagus nerve activity is a possibility *in vivo*, but our *in vitro* results indicate that the TNF-α suppressive effect of BRL-44408 is independent of a parasympathetic influence. Our present work and others' studies also show that this tremendous activation of the sympathetic and parasympathetic nervous system during sepsis is not only a result of these devastating conditions but also influences of inflammatory responses itself by regulating proinflammatory cytokines via adrenergic and cholinergic receptors.

α_2A_-ARs also have effects on the cardiovascular and nervous systems [30]–[32]. Since changes in MAP and heart rates reflect the activity of both the cardiovascular and nervous systems, we monitored MAP and heart rates during BRL-44408 administration in normal rats. Our results indicate that intravenously infusion of BRL-44408 at the dose of 2.5 mg/kg BW had no measurable effects on the MAP and heart rate. In this regard, the beneficial effect of α_2A_-AR blockade in sepsis is unlikely due to its direct effects on the cardiovascular and nervous systems.

In summary, our results suggest that hyperresponsiveness to α_2_-AR stimulation observed in sepsis is primarily due to an increase in α_2A_-AR expression in KCs. This appears to be in-part responsible for the increased proinflammatory response and ensuing organ injury in sepsis. These findings provide important feasibility information for further developing the α_2A_-AR antagonist as a new therapy for sepsis.
